# A Review on the Role and Function of Cinnabarinic Acid, a “Forgotten” Metabolite of the Kynurenine Pathway

**DOI:** 10.3390/cells13050453

**Published:** 2024-03-05

**Authors:** Kinga Gawel

**Affiliations:** Department of Experimental and Clinical Pharmacology, Medical University of Lublin, Jaczewskiego 8b Str., 20-090 Lublin, Poland; kingagawel@umlub.pl

**Keywords:** kynurenine pathway, cinnabarinic acid, aryl hydrocarbon receptor, metabotropic glutamate receptor, schizophrenia

## Abstract

In the human body, the majority of tryptophan is metabolized through the kynurenine pathway. This consists of several metabolites collectively called the kynurenines and includes, among others, kynurenic acid, L-kynurenine, or quinolinic acid. The wealth of metabolites, as well as the associated molecular targets and biological pathways, bring about a situation wherein even a slight imbalance in the kynurenine levels, both in the periphery and central nervous system, have broad consequences regarding general health. Cinnabarinic acid (CA) is the least known trace kynurenine, and its physiological and pathological roles are not widely understood. Some studies, however, indicate that it might be neuroprotective. Information on its hepatoprotective properties have also emerged, although these are pioneering studies and need to be replicated. Therefore, in this review, I aim to present and critically discuss the current knowledge on CA and its role in physiological and pathological settings to guide future studies.

## 1. Introduction

In the human body, the essential amino acid tryptophan is taken exogenously via different food products, such as, among others, chicken, turkey, milk products, tuna, cheddar cheese, etc. [1]. Melatonin, the “sleep” hormone, is one of the products of tryptophan conversion [2]. The other known pathway of tryptophan metabolism is the serotonin pathway. This is dysregulated, among others, in depression, and hence, it is targeted by antidepressant drugs [3]. Only ca. 5% of all ingested tryptophan is metabolized via the above-mentioned routes. According to the research, 95% of all tryptophan is metabolized through the kynurenine pathway [4]. This consists of several metabolites (collectively deemed the kynurenines). Some are quite well characterized, such as kynurenic acid (KYNA) [5,6], quinolinic acid (QUIN) [7,8], and (to lesser extent) l-kynurenine (L-KYN) [4]. However, there are metabolites with far less understood roles. These include xanthurenic acid and cinnabarinic acid (CA) [9].

**Figure 1:**
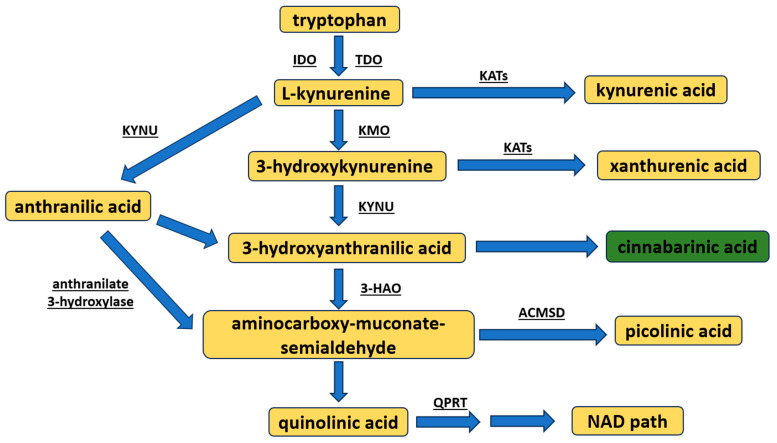
An overview of the synthesis of kynurenine pathway metabolites. The major metabolites are highlighted in yellow (note: cinnabarinic acid is accentuated in green). The following enzymes are spotlighted: 3-HAO—3-hydroxyanthranilate oxidase; ACMSD—aminocarboxy-muconate-semialdehyde decarboxylase; IDO—indoleamine-2,3-dioxygensase; KATs—kynurenine aminotransferases; KMO—kynurenine-3-monooxygenase; KYNU—kynureninase; NAD—nicotinamide adenine dinucleotide; TDO—tryptophan-2,3-dioxygenase; QPRT—quinolinate phosphoribosyltransferase.

Kynurenine pathway metabolite synthesis begins from the conversion of tryptophan to L-KYN, resulting in the formed intermediate product: N′-formylkynurenine (Figure 1) [10,11]. This occurs by way of the assistance of two enzymes: tryptophan 2,3-dioxygenase, a constitutive enzyme mostly found in the liver [10,11], and indoleamine 2,3-dioxygenase (two isoforms IDO1 or IDO2), an enzyme inducible under inflammatory conditions [12,13]. L-KYN, by the enzyme kynureninase, is almost immediately metabolized to anthranilic acid, or to KYNA, by the kynurenine aminotransferases (KAT, types I–IV with different expressions in target organs) [8]. However, L-KYN may also be transformed into 3-hydroxykynurenine via kynurenine 3-monooxygenase. In turn, 3-hydroxykynurenine is either (1) converted by kynureninase to 3-hydroxyanthranilic acid, or (2) transaminated by KATs to xanthurenic acid. Subsequently, through autoxidation, 3-hydroxyanthranilic acid may be spontaneously made into CA [14], or metabolized to the neurotoxic QUIN or picolinic acid (resulting in the intermediate product 2-aminomuconic-6-semialdehyde). QUIN is eventually converted to nicotine adenine dinucleotide (NAD) [8,15].

There are a variety of molecular targets for the kynurenines. For example, KYNA is the only known endogenous antagonist of the glycine site of the N-methyl-D-aspartate (NMDA) receptor so far. KYNA also affects the kainate and α-amino-3-hydroxy-5-methyl-4-isoxazolepropionic acid (AMPA) receptors [16,17,18]. Furthermore, some research has indicated that it is an antagonist of the nicotinic cholinergic receptor type α7 [19]; however, other studies do not confirm this observation [20]. Moreover, KYNA is known to interact with G protein-coupled receptor type 35 (GPR35) [21] and the aryl hydrocarbon receptor (AhR) [4,5]. Of note, L-KYN also binds to both of these receptors [4]. It has been shown recently that KYNA might be a ligand for adrenoceptor alpha-2B (ADRA2B) and hydroxycarboxylic acid receptor 3 (HCAR3) [5,22]. Other kynurenine pathway metabolites are known to interact with, for example, vesicular glutamate transporter (xanthurenic acid) [23], enzyme sepiapterin reductase (xanthurenic acid) [24], metabotropic glutamate receptors type II (mGlu2 and mGlu3) (xanthurenic acid) [25], and NMDA agonism (QUIN) [26]. They might also induce lipid peroxidation (QUIN) [27] and promote the production of reactive oxygen species (ROS) (3-hydroxykynurenine and 3-hydroxyanthranilic acid) [28].

The wealth of molecular targets and biological pathways associated with kynurenine activity brings about a situation wherein even slight imbalances in the kynurenine levels, both in the periphery and central nervous system (CNS), have broad consequences regarding general health. In fact, disturbances in the kynurenine pathway are implicated in the pathophysiology of cancer [6,29,30], intestine and bowel disorders [31,32], glaucoma [33,34], schizophrenia [35,36], depression [37,38], memory impairments [39,40], Huntington’s disease [41,42], Parkinson’s disease [41,43], multiple sclerosis [44,45], epilepsy [46,47], etc.

CA is the least known kynurenine. Initially described in 1957 [48], more than 60 years later, data regarding its biological role are scarce. Indeed, in 2014, Fazio et al. [49] called it the “forgotten” metabolite. Since then, more papers focused on CA have been published. Nevertheless, in the PubMed database, when typing in “cinnabarinic acid” (November 2023), only about 60 studies can be found, while in ClinicalTrials.gov, nothing is retrieved. Thus, in the current review, I aim to focus specifically on CA and its role in the context of its physiological properties and activity in pathological settings. To do so, I summarize the existing literature data on CA. Additionally, I briefly point out the most important concerns regarding the CA activity in the human body, as well as perspectives for further research.

## 2. Chemistry and Molecular Targets of Cinnabarinic Acid

### 2.1. Cinnabarinic Acid Chemistry

**Figure 2:**
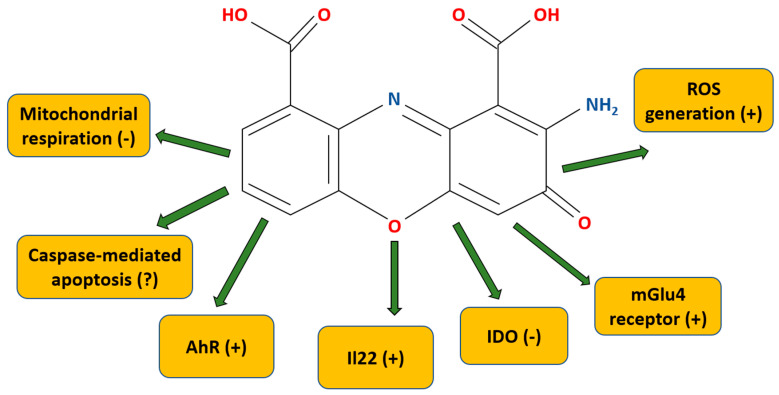
Chemical structure and overview of molecular targets and mechanisms of action of cinnabarinic acid. (+) means increases/stimulates, (−) means inhibits, (?) means contradictory data that need further investigation. For details, see text and Table 1. AhR—aryl hydrocarbon; IDO—indoleamine-2,3-dioxygenase; IL22—interleukine 22; mGlu4—metabotropic glutamate receptor type 4; ROS—reactive oxygen species.

CA (C_14_H_8_N_2_O_6_, 2-amino-3-oxophenoxazine-1,9-dicarboxylic acid, also called cinnabarinate or cinnavalininate (CAS nr 606-59-7) [50] is a member of the class of organic compounds deemed the “phenoxazines” (alternatively, the “phenoxazine chromophores”). Polycyclic aromatic compounds with a phenoxazine moiety, these are linear tricyclic systems composed of two benzene rings joined by a 1,4-oxazine ring (Figure 2). CA has a characteristic brick-brown (described also as orange-brown, red, orange-red) color, and it is primarily known as a pigment [51,52,53]. The molecular weight is 300.22 g/mol. The water solubility is low (i.e., 0.15 g/L); however, it may be dissolved in warm dimethylsulfoxide (DMSO) to make stock (25 mM) [50].

Very recently, Gómez-Piñeiro et al. [54] provided new data about the metabolism and stability of CA under physiological conditions, with CA’s stability being studied under varied conditions (solvent, aerobic/anaerobic conditions, pH, temperature). In the initial experiment, CA was dissolved in DMSO, methanol, and acetonitrile to methanol 1:1 at 37 °C. Regardless of the solvent, under such conditions, the CA was stable up to 8 h. When an equivalent volume of water was added, the stability was lost. Also, with lower pH, a greater stability was indicated. Under anaerobic conditions, the half-life for CA in phosphate buffer saline (37 °C) was found to be the highest at ca. 8.7 h. In other test conditions, various reductant compounds were utilized. All, though to different extents, substantially shortened the CA half-life in the experimental medium. CA, when dissolved in phosphate buffer saline (37 °C) and added to rat liver microsomes, had a half-life of ca. 3.9 h (note: its sub-cellular faction is mostly composed of cytochrome P450 (CYP) enzymes). In addition, when nicotinamide adenine dinucleotide phosphate (NADPH), a natural co-factor of CYP enzymes, was added to the mixture, it disappeared within less than 3 min, and it was discovered that the hem-active site of CYPs is involved in this process. When a CYP inhibitor was added to the reaction mixture, it prolonged the CA stability half-time. Next, the authors demonstrated that other products (i.e., dihydrocinnabarinic acid) may be produced during the decomposition process and that light (un-irradiated conditions) does not affect the CA stability.

This work is very valuable because it provides knowledge about the CA stability. Such data must be considered when interpreting results; for example, negative outcomes may not necessarily come about due to the lack of CA activity but because of CA solution instability, as well as the presence of other metabolic products if reductants are added to the solution.

### 2.2. Cinnabarinic Acid Synthesis

In the human body, CA, formed by the non-enzymatic condensation of two molecules of 3-hydroxyanthranilic acid, is a byproduct of the kynurenine pathway. There are, however, reports that CA is also produced in the orange dead leaf butterfly *Kallima inachus*, where its decrease triggers the larva-to-pupa transition [55]. CA has also been found deposited in the fruiting bodies of saprophytic white rod fungi from the *Polyporaceae* family in, among others, *Pycnoporus cinnabarinus* and *Pycnoporus sanguineus* [56,57,58,59] and in southern cinnabar polypore (*Trametes coccinea*) [60]. In the fungus *Pycnoporus cinnabarinus*, CA is created through the laccase-mediated oxidation of 3-hydroxanthranilic acid [57,59], and it is the presence of CA that gives these mushrooms their characteristic brick-brown color. CA is considered a natural pigment, and its isolation from *Pycnoporus cinnabarinus* culture media at the laboratory scale has been described [60].

It has been shown in laboratory conditions that laccase [57,61] (which also catalyzes CA synthesis in fungi, as mentioned above), catalase [62,63], tyrosinase [64], superoxide dismutase (SOD) [14,62,65], horseradish peroxidase [62,66], and myeloperoxidase [62] may catalyze the conversion of 3-hydroxyanthranilic acid to CA.

In the Malphigian tubules, but not in the hemolymph of silkworm *Bombyx mori*, 3-hydroxyanthranilic acid is oxidized to CA (in the presence of manganese ions) [67]. Moreover, the synthesis of CA has been found to take place in the leaves of *Tecoma stans* [68]. Because the other metabolites of the kynurenine pathway, such as KYNA [5,69,70,71,72,73] and, to lesser extent, also L-KYN [4], are found in different plants, beverages, and food products, it would be interesting to test whether certain food products here-to-fore not documented might also be good sources of CA.

### 2.3. Receptors and Molecular Mechanisms of Action

CA is the only product of the kynurenine pathway capable of interacting with the mGlu4 receptor (for a summary of the CA mechanisms of action, see Table 1) [74]. The mGlu4 receptor is a Gi/Go protein-coupled receptor (GPCR). These are located presynaptically and impede neurotransmitter release [75]. CA does not display activity against other mGlu receptor subtypes [74]. CA binds in the glutamate-binding pocket, in the extracellular “Venus flytrap” domain, and, specifically, within the orthosteric site of the mGlu4 receptor located there. According to Fazio et al. [74], CA has partial agonist activity towards mGlu4 receptors. In related work, using cultured granule cells, which are known to release glutamate, the authors demonstrated that CA inhibits the formation of cyclic AMP. Of note, the researchers showed that when mGlu4 receptors are knocked out, high doses of CA still exerted activity. Therefore, they suggested that CA may have off-target effects [74].

Pasceri et al. [76] argued that CA is an IDO inhibitor with the K_i_ value at 326 nM. The CA IC_50_ is, according to their research, equal to 0.46 μM (the percentage of enzyme activity remaining for 0.1, 1, or 10 μM being 71, 23, and 11%, respectively). This observation agrees with Carr et al. [77], who demonstrated CA-mediated IDO inhibition, but with the IC_50_ being ca. 2 μM.

In 2014, Lowe et al. [78] found a new mechanism of CA activity. In their work, human peripheral blood mononuclear cells (PBMCs) stimulated with antibodies against CD3 and CD8 in vitro were exposed to a board of kynurenine pathway metabolites (i.e., 3-hydroxykynurenine, 3-hydroxyanthranilic acid, QUIN, and picolinic acid). Of these, 3-hydroxykynurenine and 3-hydroxyanthranilic acid (but not QUIN or picolinic acid) were able to promote IL22 release in CD4^+^ T cells. The upregulation of IL22 in CD4^+^ cells, but not in CD8^+^ T cells, was AhR-mediated, as the blocking of AhR by the potent, selective antagonist CH-223191 prevented the production of IL22. AhR, being a ligand-mediated transcription factor that is expressed ubiquitously in human tissues, is mostly involved in common metabolic functions [79]; however, its activation has an important role within several pathological processes. These include inflammation and carcinogenesis [5,6,80], and AhR is also responsible for the removal of toxic compounds (e.g., drugs or environmental toxins) [81]. Still, as noted in a series of complementary experiments, 3-hydroxyanthranilic acid itself is not an AhR ligand but is the direct precursor of the endogenous AhR ligand, CA. In the utilized experiments, the CA activity was found to be linked to the upregulation of IL22 (but not IL17) in CD4^+^ T cells, and AhR was seen as being concentration-dependent. CA also was also discovered to induce the in vitro and in vivo expression of *cyp1a1*, a downstream AhR-mediated gene [78].

When compared with other kynurenine pathway AhR ligands (i.e., KYNA and L-KYN), CA was found to be less effective in the upregulation of *cyp1a1*. However, research indicated that it brought about IL22 levels higher than the aforementioned two metabolites, and this effect was not *cyp1a1*-dependent. Other research undertaken demonstrated that human PBMCs can produce CA when challenged with lipopolysaccharide (LPS), a bacterial toxin that is known to induce overt inflammation, or with inflammatory cytokine interferon γ (IFNγ). Herein, mouse naïve CD4^+^ T cells produced CA only when the fungal enzyme, laccase, was present in the media. As such, this study clearly revealed a new molecular target for CA [78].

CA was found to inhibit state 3 mitochondrial respiration [82]. What is more, 3-hydroxyanthranilic acid was discovered to be rapidly oxidized by cytochrome c to CA. In the study, rat liver and beef heart mitochondria were incubated in CA concomitant with various substrates (e.g., α-ketoglutarate, malate, isocitrate, pyruvate, or glutamate). The results of the work demonstrated that a one-hundred-fold excess of glutathione in the incubation medium did not protect the rat liver and beef heart mitochondria from CA inhibition. CA was discovered to be at least twenty times more effective than 3-hydroxyanthranilic acid in inhibiting rat liver mitochondrial respiration. A 1 mM concentration of 3-hydroxyanthranilic acid decreased glutamate, malate, pyruvate, and isocitrate oxidation by around 40–70%, with 50 μM of CA giving a similar effect. When α-ketoglutarate was used as an oxidized substrate, this effect was even more evident. Here, half-maximal inhibition was seen for 250 μM of 3-hydroxyanthranilic acid and 2 μM of CA. Because high amounts of 3-hydroxyanthranilic acid were found to be excreted in the urine of an individual with bladder tumors [83], the author of this work also put forward a very interesting hypothesis, although so far unverified, that the CA interaction with mitochondria is responsible for bladder tumor induction [82].

Eventually, Zollner’s observation regarding the CA-induced inhibition of mitochondrial respiration was confirmed by Nagamura et al. [84], who, in turn, indicated that a 10 μM concentration of CA completely inhibited the mitochondrial respiration in an injured liver, thereby aggravating the symptoms of injury.

There is data that CA is able to generate ROS and induce caspase 3-mediated apoptosis in thymocytes [85]. For the latter, CA is more than ten times more efficient than its precursor, 3-hydroxyanthranilic acid. According to the results of the experimental work, the intracellular ROS generation after CA application was very rapid—taking place as early as 15 min after the CA application, and returning to the control level after 4 h. In contrast, ROS generation by 3-hydroxyanthranilic acid increased gradually up to 4 h. Under further experimentation, it was found that the process of CA-mediated ROS induction was inhibited by SOD, as well as catalase, or a mixture of the two. Likewise, 40% of the mitochondrial membrane potential was disrupted within the first 15 min upon CA administration and was kept constant afterwards.

The above data are contrary, however, to those of Joshi et al. [86,87], who revealed in their work that, in mice, CA decreased the caspase-3 overexpression in hepatocytes and caspae-3/7 overactivity in liver homogenates after incubation with ethanol. Thus, this mechanism needs further verification.

**Table 1:** Overview of proposed molecular targets or mechanisms of action for CA. AhR—aryl hydrocarbon receptor; CA—cinnabarinic acid; ca.—circa (about); IC_50_—half-maximal inhibitory concentration; IDO—indoleamine-2,3-dioxygenase; IL22—interleukin 22; K_i_—the inhibitor constant; mGlu4—metabotropic glutamate receptor type 4; ROS—reactive oxygen species.

Molecular Target/Mechanism	CA Activity (Summary of Data Found in References)	References
IDO inhibition	IC_50_ ≈ 2 μM	[77]
	K_i_ value at 326 nM IC_50_ was equal to 0.46 μM	[76]
mGlu4 receptor orthosteric agonist	100 μM CA increases [3H]Ins5 formation by ca. 35% (it is 5× less efficacious than the full mGlu4 agonist ACPT-I); CA binds within the glutamate-binding pocket.	[74]
AhR agonist; production of IL22	CA increases (1 μM) the production of IL22 in human and mouse CD4+ T cells through AhR (the blocking of this receptor prevents the IL22 increase).	[78]
Inhibition of mitochondrial respiration	Complete inhibition at 5 μM; 0.5 μM of CA leads to 50% inhibition of state III respiration.	[84]
	CA is at least 20× more efficient at inhibition than 3-hydroxyanthranilic acid.	[82]
ROS generation	CA brings about the rapid induction of ROS generation (ca. 15 min, with return to the control level after 4 h).	[85]
Apoptosis	Induction: CA holds at least 10× higher apoptosis-inducing properties when compared with 3-hydroxyanthranilic acid. The caspase-3 activity is upregulated in the thymocytes within 6 h after simulation with 30 μM of CA.	[85]
	Antiapoptotic properties: CA alleviates caspase-3 or caspase-3/7 upregulation in ethanol-treated hepatocytes/liver lysates. No direct effect of CA itself is indicated.	[86,87]

## 3. In Vivo Studies

Ulivieri et al. [88] assessed the levels of CA in prefrontal cortex (PFC) samples of schizophrenic and healthy-matched control patients (for a summary of the concentrations of CA in the tissues and body fluids, see Table 2). The CA level was reduced in the schizophrenic patients, compared to the controls, and there was no correlation between the CA levels in the PFCs and the age of the patients or their sex. Neither the duration of treatment nor the type (classical vs. atypical) or duration of antipsychotic drug regimen showed correlation. The CA level seemed to be stable, as there was lack of correlation between the CA content in the PFC and post-mortem intervals.

In related work [88], an intraperitoneal (*ip*) injection of 0.25 mg/kg of CA into experimental mice caused a peak in the CA levels in the sera after 30 min, and in the mouse cortex and cerebellum, it caused a peak after 1 h (the method used was sensitive enough to detect the CA level in picograms/gram of tissue also in the sera and brains of control counterparts). The level of CA was relatively stable in the cortex and cerebellum samples up to 12 h after injection, and the blood–brain barrier permeability of CA was confirmed immunohistologically. The level of CA increased when LPS, an inflammation inducer, was given.

Bearing this in mind, the authors of [88] analyzed the effect of the systemic administration of CA on psychotic-like behavior in mice. CA, given in a range of doses (0.125, 0.25, 0.5, 1, 5, or 20 mg/kg, *ip*), except for 20 mg/kg, significantly reduced the MK-801-induced hyperlocomotion (mouse model of psychotic-like behavior) without any influence on the basic animal activity. In the pre-pulse inhibition test in rats, CA was found to reverse the inhibitory activity of MK-801 only at the lowest dose (i.e., 0.25 mg/kg) (but not at 0.125 or 0.75 mg/kg). Likewise, this dose-dependent trend was observed in the novel-object recognition test when memory was disturbed by MK-801 administration. Of note, pretreatment with CA (0.25 and 20 mg/kg) reversed the social interaction disturbances in the MK-801-treated animals. This work demonstrated that the antipsychotic activity of low doses of CA (from 0.125 to 0.5 mg/kg) is mediated through mGlu4 receptors because the systemic administration of CA did not prevent MK-801-induced hyperlocomotion in the *mGlu4^−/−^* mice.

The authors of [88] also revealed that 0.5 mg/kg of CA prevented the release of glutamate from the PFC after MK-801 administration. Such studies continued in vitro. Herein, the CA did not inhibit NMDA receptors, though it did inhibit glutamate (through the activation of presynaptic mGlu4 receptors) and, to a lesser extent, the neurotransmitter gamma-aminobutyric acid (GABA) release.

This comprehensive, multidirectional research has provided new, important findings about the role of CA [88]. Moreover, it confirmed the already existing data that CA is brain-barrier-permeable. It also revealed the actual levels of CA in sera (mice) and brain samples (mice and humans). Additionally, for the first time, CA was demonstrated to be implicated in the pathophysiology of schizophrenia.

More recently, Shilov et al. [89] measured the concentration of CA and 3-hydroxyanthranilic acid in the blood of 23 schizophrenic patients (depressive–delusional type) at two time points: before and after the implementation of drug treatment. These individuals were recruited when the exacerbation of the disease’s symptoms occurred. The patients, during their treatment, were administered different antipsychotics. The PANSS (Positive and Negative Symptom Rating Scale) was applied to assess the clinical schizophrenic symptoms, and the HDR (Hamilton Scale) was employed to assess the depressive symptoms. The mean concentration for CA in the blood samples before the implementation of treatment was found to be 11.26 nmol/L, whereas the CA concentration after treatment was 8.03 nmol/L. Furthermore, the mean concentration of 3-hydroxyanthranilic acid was 14.95 nmol/L, and it was 20.05 nmol/L before and after treatment. The authors calculated the sum of both metabolites (CA + 3-hydroxyanthranilic acid), as they argued that it may better reflect the state of the kynurenine pathway. Inverse statistical significance was shown between the sum of both metabolites and CA before treatment and the PANSS score after treatment. No correlation was discovered between the metabolites separately, nor in their sum between and after treatment. This study, however, possesses several limitations: (1) first of all, the methodology for the CA measurement contains several gaps, which may make this study difficult to replicate; (2) it is unclear when exactly the blood samples were collected (after, e.g., 12 h, a few days, or a few months after treatment implementation); and (3) the correlation between specific drug treatments and CA is not provided. Taking this into account, one can conclude that it is too early to point towards any final opinion about the relevance of Shilov’s findings [89].

Alterations of kynurenine pathway metabolites, including CA, were investigated in patients with autism spectrum disorder (ASD) [90]. Accordingly, the levels of CA and stanniocalcin 2 were higher in individuals with ASD than in the controls (note that *Stc2* encodes stanniocalcin 2, a glycoprotein involved in cell metabolism, inflammation, apoptosis, calcium homeostasis, etc.). In addition, IL22 level (regulated by AhR) was higher in individuals with ASD than in the controls. The researchers noted a very strong positive correlation between CA and IL22 only in individuals with ASD. In summary, this study reveals that, in patients with ASD, higher activity of IDO is observed, and the synthesis of metabolites is shifted towards CA, which activates AhR and its downstream target *Stc2*. Undoubtedly, this comprehensive and elegant study is of significant value and provides evidence that AhR blockage may be a target in the treatment of ASD [90].

The activity of systemically (*ip*) administered CA was investigated by Notartomaso et al. [91] in preclinical models of acute inflammatory (formalin model) and neuropathic pain (chronic constriction injury (CCI)). The authors investigated the hypothesis that CA, as a mGlu4 agonist, has analgesic activity [92,93]. In the formalin test, low doses of CA (0.125 or 0.25 mg/kg), but not high doses (0.5 or 3 mg/kg), reduced the nocifensive behavior, compared to the control mice, and the CA-induced effect in the formalin test was mediated through the activation of mGlu4 receptors but not AhR.

Additionally, it was shown that an acute administration of CA (0.25 mg/kg, *ip*) is analgesic in CCI mice. Of note, chronic administration did not induce analgesic activity, which means that the development of tolerance occurred, but it also did not appear when CA was co-administered with CH223191, an AhR antagonist. Electrophysiological studies indicated that CA and CH223191 given together, but not singly, did, however, reduce the activity of the nociceptive neurons.

Taken together, this paper showed for the first time the analgesic properties of CA in only very low dosages [91]. This effect may be related (as the authors suggest) to rapid receptor desensitization or to the recruiting of other molecules, the activity of which counterbalances CA-induced analgesia. This study has scientific value, as the development of tolerance to CA-mediated analgesia excludes this compound for the treatment of chronic pain, at least when given alone.

Fazio et al. [49] investigated the effect of the systemic administration of CA (doses from 0.1 to 10 mg/kg, *ip*) in mice with experimentally-induced autoimmune encephalomyelitis (EAE). When treatment with CA (10 mg/kg, *ip*) after immunization commenced after a 7-day delay, partial protection was observed. The same was noted when treatment with CA was limited to 21 days post- immunization. Furthermore, the lowest and highest doses of CA given once daily for 35 days after immunization completely suppressed the clinical symptoms of EAE. Additional studies also demonstrated that a 35-day-long chronic treatment regimen with CA contracted the demyelination processes in the mouse spinal cords and minimized the accumulation of migratory cells in the mouse brains and spinal cords. Beyond the aforementioned, the cytokine profiling of CD4^+^ T cells isolated from lymph nodes or from brain-infiltrating leukocytes indicated that CA-pretreatment + immunized mice decreased the IFNγ and IL-17 levels compared to immunized-only animals. In contrast, the level of TGF-β was higher in the CA-treated animals both in the lymph nodes and brain-infiltrating leukocytes, whereas the level of anti-inflammatory IL-10 was upregulated only in the lymph nodes, but its level did not differ between groups in the brain-infiltrating leukocytes. The authors hypothesized that CA administration may trigger the synthesis of IDO (induced in inflammatory conditions) and kynureninase enzymes (which covert 3-hydroxykynurenine to the ultimate precursor of CA (i.e., 3-hydroxyanthranilic acid)). This was confirmed utilizing purified splenocytes at 20 days post-immunization. Moreover, the experimenters revealed that endogenous CA synthesis requires antigen-specific stimulation. In complementing their observations, CA (10 mg/kg, *ip*) was administered to immunized *mGlu4^−/−^* mice and was seen to reduce (to some extent) the clinical scores in *mGlu4^−/−^* mice. This suggests that the protective properties of CA are mediated by additional mechanisms apart from *mGlu4* activation.

Considering the role of AhR, which is the ligand-activated transcription factor responsible for xenobiotic metabolism, it would be useful to test whether these receptors are implicated in CA-mediated protection in the multiple sclerosis model in mice. Collectively, this study clearly shows the positive activity of CA towards neuroinflammation [49].

The activity of CA (5 or 10 mg/kg, *ip*), a key ingredient of pu-erh tea, was also investigated in a mouse model of circadian rhythm-related obesity [94]. Herein, CA was seen to decrease the food intake and did slow the weight gain of mice that were exposed to circadian rhythm disturbances. In addition, it inhibited the deposition of liver fat, reduced inflammation in the liver, and diminished the white-fat deposition in the epididymis. The upregulation of the mGlu4 receptor was, moreover, seen in the CA-treated mice, and CA intake had a positive effect upon the gut microbial composition, as an increase in the *Lactobacillus* and *Eubacterium* abundance was revealed [94]. It is worth noting that Joshi et al. [95] did not note the influence of CA administration on the body weights (12 mg/kg, *ip* for 11 days) of normal (non-obese) mice. Thus, it cannot be excluded that the effect of CA is only seen in obesity. Our research, however, indicated that a significant reduction in the body weight gain of rats postnatally exposed to KYNA supplementation came about. This was without changes in the total body surface and bone mineral density [96].

Joshi et al., in a series of papers, analyzed the cytoprotective effects of CA against apoptosis induced by endoplasmic reticulum stress, oxidative stress, and alcohol insult (acute and chronic) and in non-alcoholic fatty liver disease [86,87,95,97]. They revealed that the CA-driven cytoprotecting activity is associated with the activation of AhR. This effect was found to be specifically linked to the upregulation of its downstream gene *Stc2.* As mentioned earlier, mice given CA for 11 days (12 mg/kg, *ip*) did gain weight similar to their control counterparts [95].

The effect of CA on developing zebrafish (*Danio rerio*) was assessed by Majewski et al. [98]. Herein, the incubation of fish with 30 μm of CA from 24 to 96 h post-fertilization (hpf) induced severe morphological abnormalities: among others, notochord anomalies, small eyes, severe heart and yolk sac oedema, and jaw underdevelopment. When given acutely, 180 μm of CA also increased the heart rate compared to control larvae. The same phenotype was seen when embryos were incubated in CA (concentrations of 5, 10, and 50 μm) between 24–48 hpf and 24–72 hpf. In addition, Majewski et al. [98] observed tremors and convulsions in larvae incubated in CA from 24 to 124 hpf.

The effect of CA was also investigated in larval zebrafish by Lowe et al. [78] in the context of *cyp1a1* upregulation. In their experimental settings, a TL zebrafish strain was used (compared to the Tubingen strain in Majewski et al. [98]), and the concentrations administered were higher (100 μm, but the fish were incubated for only 6 h). Both papers [78,98], however, lack detailed information on how the CA solution was prepared.

Compared especially to KYNA, based on the data of Majewski et al. [98], CA seems to be teratogenic. However, this is a single study that needs replication to make a proper conclusion, considering that, for example, the observations for L-KYN of Majewski et al. [98] are not in agreement with those of Marszalek-Grabska et al. [99].

**Table 2:** Content of CA in tissues and body fluids of human and experimental animals. ASD—autism spectrum disorder; CA—cinnabarinic acid; ca.—circa (about); *ip*—intraperitoneally; HPLC-MS/MS—high-performance liquid chromatography–mass spectrometry; LC-MS/MS—liquid chromatography–mass spectrometry; LPS—lipopolysaccharide; SEM—standard error of the mean; PFC—prefrontal cortex; UPLC-MS/MS—ultra-performance liquid chromatography–mass spectrometry.

Species	Group	Content of CA in Tissues/Body Fluids [Value in pM Recalculated for Comparison between Studies]	Comment(s) (If Applicable)	Method for Quantification	References
Human	23 adult individuals with schizophrenia (16 males and 7 females) and 26 non-schizophrenic patients	PFCs of healthy-matched control patients: mean ± SEM: 35.92 ± 1.99 pg/g tissue [119.64 ± 6.62 pM]	Reduced levels of CA in PFCs of schizophrenic patients vs. controls No correlation between CA levels in PFC and age of patients or their sex	UPLC-MS/MS	[88]
		PFCs of schizophrenic patients: mean ± SEM: 22.61 ± 1.65 pg/g tissue [75.31 ± 5.49 pM]			
Human	23 female, adult patients with schizophrenia	Plasma (before treatment with antipsychotics): mean: 11.26 nmol/L (min. 3.03 nmol/L–max. 38.31 nmol/L) [mean: 11,750 pM, min. 3160 pM–max 40,030 pM]	No statistical difference between groups	HPLC-MS/MS	[89]
		Plasma (after treatment with antipsychotics): mean: 8.03 nmol/L (min. 2.49 nmol/L–max. 45.30 nmol/L) [mean: 8390 pM, min. 2600 pM–max. 47,360 pM]			
Human	Adult patients with ASD (90 ASD patients and 104 controls)	Plasma (healthy-matched control patients): ca. 0.25 nM (mean) [250 pM]	Detailed concentrations not provided CA levels higher in ASD patients	LC-MS/MS	[90]
		Plasma (ASD group): ca. 0.75 nM (mean) [750 pM]			
Sprague-Dawley adult, male rats	Controls	Lungs: ca. 60 pg/mg [199.85 pM] tissue Kidney, liver, and spleen: 7–10 pg/mg [23.31–33.30 pM] tissue Brain: below detection limit Striatal dialysate: below detection limit	-	HPLC-MS/MS	[74]
	After LPS challenge	Lungs: below detection limit Kidney: 133 pg/mg [443.0 pM] tissue Spleen: 36 pg/mg [119.91 pM] tissue Brain: 160 pg/mg [532.94 pM] tissue Striatal dialysate: below detection limit	-		
C57BL/6 adult, male mice	Experimentally evoked autoimmuneencephalomyelitis	Cerebrospinal fluid: below detection limit	-	HPLC-MS/MS	[74]
C57BL/6 adult, male mice	Controls	Serum: ca. 4 ng/mL [13,320 pM] Cortex: ca. 20 pg/g [66.61 pM] tissue Cerebellum: ca. 14 pg/g [46.63 pM] tissue	Detailed concentrations not provided	UPLC-MS/MS	[88]
	CA (0.25mg/kg, *ip*) acutely injected	Serum: ca. 30 ng/mL [99,920 pM] after 0.5 h, ca. 6 ng/mL [19,984 pM] after 1 h, and ca. 4 ng/mL [13,320 pM] after 3 h post-injection Cortex: below 500 pg/g [1665 pM] tissue after 0.5 h, above 1000 pg/g tissue after 1 h, ca. 1000 pg/g [3330 pM] tissue after 2 h, and from 3 to 12 h post-injection at relatively stable level of ca. 500 pg/g [1665 pM] tissue Cerebellum: ca. 200 pg/g [666.17 pM] tissue after 0.5 h, a little below 600 pg/g [1998.51 pM] tissue after 1 h, ca. 400 pg/g [1332.34 pM] tissue from 3 to 4 h post-injection; above 200 pg/g [666.17 pM] tissue at 12 h post-injection			
Female mice (*Stc2^+/+^*, C57BL/6 background)	Controls	Liver: ca. 10 pg/mg [33.30 pM] tissue Serum: -	Detected in serum but impossible to specify concentration from figure (lack of detailed information in the text)	HPLC-MS/MS	[94]

## 4. In Vitro Studies

Fazio et al. [74] analyzed the effect of CA on the viability of cultured cortical neurons challenged with excitotoxic NMDA. Herein, CA itself in the range of the tested concentrations (from 10 to 300 μM) did affect the viability of the neurons either after 10 min, or after 24 h of incubation. In contrast, in concentrations above 30 μM, it did protect the neurons against death after the application of NMDA. There was not, however, a concentration-dependent effect, as all doses above 30 μM protected the neurons in equal potency. Furthermore, in the cultures prepared from *mGlu4^−/−^* mice, neuroprotection was evident, but it was lower compared to the wild-type cultures.

To strengthen their findings, the authors of [74] infused CA (50 nmol/0.5 μL) to the globus pallidus to establish whether CA is protective against the MPTP-induced damage of the dopaminergic neurons in the nigrostriatal pathway (1-methyl-4-phenyl-1,2,3,6-tetrahydropyridine (MPTP) is a toxin used to induce Parkinson’s-like symptoms). In their experimental setup, they showed that CA is neuroprotective.

There are data on the influence of antiseizure medications (ASMs) on the release of KYNA, though this seems to be a drug-dependent activity [100,101,102]. Likewise, the effect of levetiracetam and zonisamide on the release of, among others, CA was analyzed using rat cortical astrocyte cultures [103,104]. In the latter work, Fukuyama and Okada [104] showed that chronic incubation with inflammatory cytokine IFNγ did affect the astroglial release of KYNA and L-KYN, whereas the CA level was below the detection limit. When 3-hydroxykynurenine was added to the reaction mixture (medium + IFNγ, chronic incubation for 7 days), CA was detected. A concentration-dependent effect of IFNγ was also observed: for 100 U/mL of IFNγ, the level of CA was around 10 nM, and this IFNγ concentration was the same as the level of xanthurenic acid but was lower than that of the QUIN level (a little above 15 nM). Levetiracetam, a new-generation ASM, when added to a medium with 3-hydroxykynurenine, did not affect the CA concentration itself, but when given in combination with IFNγ, its level doubled for concentrations of 10 and 30 μM. The acute administration of excitotoxic AMPA and adenophostin A (agonist at the IP3 receptors) to a medium increased the CA level, but when levetiracetam was added, this effect was reversed. Together with the data obtained by the same research group for zonismide (another ASM) [104], one may assume that CA might be implicated in the mechanism of action of ASMs, though more studies are needed (especially in vivo) to make any final conclusions.

According to several studies, liquid culture filtrate obtained from *Pycnoporus cinnabarinus* [57], *Trametes coccinea* [105], and *Pycnoporus sanguineus* SYBC-L7 [52] shows antimicrobial activity. The presence of CA is largely responsible for this effect. In the course of such work, the lowest maximal inhibitory concentration was obtained for bacteria from the *Streptococcus* genus (group B, D, F, G), though CA was also effective towards the *Staphylococcus* genus, *Bacillus* genus, *Pseudomonas aeruginosa*, *Klebsiella pneumoniae*, *Escherichia coli*, and *Salmonella enteriditis* [57]. In general, the inhibition was seen to be greater for Gram-positive rather than Gram-negative bacteria [57,105]. Beyond the aforementioned, the formation of bacterial biofilms (*Bacillus cereus* and *Bacillus subtilis*) was seen to be inhibited by CA [105]. Considering that CA occurs naturally and may be produced on preparative scale, one may conclude it might be worth investigating CA as a new antimicrobial agent.

## 5. Conclusions and Future Perspectives

Compared to, e.g., KYNA, the biological role of CA is widely unknown. As an endogenous byproduct of the kynurenine pathway, CA seems to have an important function in several physiological circumstances. One may assume that the little interest in this metabolite is because there are difficulties in finding it in biological material. Even in the same papers, the authors acknowledged that it was or was not detected in body fluids [74,95] or in samples [103,104]. Its content in serum is also much smaller than that of KYNA (nM vs. μM concentrations) [5]. Therefore, it is not without reason that it is called “trace’’ kynurenine [74,88]. One may not exclude, however, that it is not a lack of scientific community interest in CA but rather the technical difficulties for its measurement that have brought about the situation wherein it is less investigated than the other kynurenine pathway metabolites. The latest study of Gómez-Piñeiro et al. [54] also shows that CA is unstable under physiological conditions, and that, in the presence of reductants, it readily metabolizes to other products. Thus, this may also be another reason that its levels are below the detection limit. The available data, however, indicate that CA seems to be well worth investigating, though the first step is overcoming obstacles related to its measurement in biological samples.

There are very few data in the literature regarding the biological function of CA. Mostly, these are single papers; thus, even if of high scientific value, it is not possible to confirm the findings, as comparative studies have not been undertaken in independent experiments. Furthermore, there are also some papers that are contradictory. One can assume that, at this stage of the knowledge, it is impossible to give any final conclusions about the role of CA in the human body, though it seems that, similar to KYNA, CA may have neuroprotective properties [49,74,88]. It is also possible that it is hepatoprotective, but this must be confirmed by research groups other than Joshi et al. [86,87,94,96]. At this stage of the knowledge, it is not possible to directly compare CA to KYNA, as too many gaps in the current knowledge exist for CA (no data about its role e.g., in epilepsy, Alzheimer’s disease, cancer, etc.), and there is only a single study demonstrating the existence of a role in ASD and schizophrenia.

When practical methods for determining this metabolite are developed, CA’s effects in health and disease will be an unlimited topic for research. So far, the role of CA has been investigated in autism, schizophrenia, and pain, and these studies must be replicated. Studies on the role of CA activity regarding other diseases, like depression, epilepsy, anxiety, neurodegenerative diseases, etc., are warranted. Similarly, the role of CA in the context of inflammation-related diseases might be worth investigating. Moreover, there is a lack of data on how and whether CA affects the microbiome and gut–brain axis, and it is unclear what food products may be a source of CA. There is a lack of knowledge on how exactly CA synthesis relates to the synthesis of other kynurenine pathway metabolites, and this gap has to be filled. When evidence is related to tumors, metabolic changes are noted, and the CA activity can vary. Thus, its activity in various forms of cancer must be thoroughly assessed. It is reasonable, therefore, to expect that the coming years might at least partially address these issues.

## Data Availability

Not applicable.
